# The value of multimodality imaging in diagnosis and treatment of cardiac lipoma

**DOI:** 10.1186/s12880-021-00603-6

**Published:** 2021-04-15

**Authors:** Shenglei Shu, Hongliang Yuan, Xiangchuang Kong, Jiazheng Wang, Jing Wang, Chuansheng Zheng

**Affiliations:** 1 Department of Radiology, Union Hospital, Tongji Medical College, Huazhong University of Science and Technology, No.1277 Jiefang Ave, Wuhan, 430022 China; 2 Hubei Province Key Laboratory of Molecular Imaging, Wuhan, China; 3 Department of Ultrasound, Union Hospital, Tongji Medical College, Huazhong University of Science and Technology, Wuhan, China; 4 Clinical and Technical Solutions, Philips Healthcare, Beijing, China

**Keywords:** Cardiac lipoma, Noninvasive imaging, Magnetic resonance imaging, Computed tomography

## Abstract

**Background:**

Cardiac lipoma is a rare primary tumor in the heart and pericardium. Multimodality imaging methods, especially magnetic resonance imaging (MRI), are crucial in detecting and diagnosing cardiac lipomas. Besides, they are of significant importance in management of cardiac lipomas. The aim of this study was to evaluate the value of multimodality imaging methods in diagnosing and treatment of cardiac lipoma by describing a series of cases of cardiac lipoma.

**Materials and methods:**

Data of patients with cardiac lipoma at a local institution were retrospectively collected. Their imaging findings on echocardiography, computed tomography (CT), and cardiac MRI and clinical management were described in detail.

**Results:**

12 patients with cardiac lipoma were retrospectively included with thirteen lipomas found within heart and pericardium. Two patients’ lipoma were symptomatic, while lipomas in other 10 patients were found incidentally. Most lipomas were sensitively detected with echocardiography. Accurate diagnoses were achieved with CT and MRI in all cases. Surgical resection was performed in one symptomatic patient due to the obstruction of the left ventricular outflow tract, while the removal of pericardial lipoma in another symptomatic patient was not possible due to diffuse myocardial infiltration observed in MRI. Based on MRI findings, two patients without clinical symptoms also underwent surgery to prevent the risk of detachment of ventricular lipoma with a narrow pedicle in one patient and potential further thinning of the myocardium by pericardial lipoma growth in another patient.

**Conclusions:**

Cardiac lipoma could be sensitively detected and accurately diagnosed with multiple noninvasive imaging tools. Comprehensive evaluation with multimodality imaging methods should also be conducted for better management planning and follow-up in all patients.

## Introduction

Primary cardiac tumors are uncommon, of which cardiac lipoma is even rarer [1]. They are usually asymptomatic and found incidentally during surgery or autopsy [2]. However, they may cause clinical presentations varying from mild chest discomfort to even sudden death [3, 4]. Nowadays, the detection and diagnosis of cardiac lipoma are highly dependent on noninvasive imaging methods including echocardiography, computed tomography (CT), and magnetic resonance imaging (MRI) [5]. Besides providing an accurate diagnosis, findings from noninvasive imaging methods may also modulate the management of cardiac lipomas aside from clinical presentation. This study aimed to present cardiac lipoma cases diagnosed with noninvasive cardiac imaging methods. The clinical data and imaging findings of cardiac lipomas were described with special focus on the effect of cardiac imaging on clinical management.

## Materials and methods

Data of patients with cardiac lipomas were retrospectively collected from a local institution. Patients with cardiac lipomas confirmed by pathology were included by searching pathological records of the hospital. Besides, patients diagnosed with cardiac lipoma by MRI were also included because MRI was sufficient to diagnose lipoma [6]. Patients with lipomatous hyperplasia of the interatrial septum, but not true cardiac lipoma, were excluded [7].

For each patient, the clinical data including sex, age at the time of diagnosis, onset symptoms, and management of the cardiac lipoma were collected from medical records. Follow-up was done for all patients through phone call at the time of writing. The morphological features of cardiac lipomas, including number, location, shape, and size, were obtained from multimodality imaging data, especially cross-sectional images. In addition, the echogenic features of cardiac lipomas, including echo intensity, homogeneity, and hemodynamic abnormality related to cardiac lipoma, were reviewed by two sonographers with more than 10 years of experience in echocardiography. The imaging characteristics of cardiac lipomas in CT and MRI, including attenuation value, signal intensity, and enhancement features (if contrast scanning was used), were also observed and documented by two radiologists with more than 5 years of experience in cardiovascular imaging. The disagreement between two observers was solved by consultation. Our study complied with the Declaration of Helsinki and informed consent was waived due to the retrospective nature of the study by ethical committee of Tongji Medical College of Huazhong University of Science and Technology.

## Results

From February 2013 to December 2019, 12 patients diagnosed with cardiac lipomas were included in the study. The clinical demographics of included patients are shown in Table 1 arranged by order of date of admission. The patients’ age ranged from 18 to 82 (median age, 54) years, with a male predominance among included patients (8 males, 66.7%). The diagnosis of cardiac lipoma was achieved with cardiac MRI in all cases, while only three of them were confirmed by pathology after surgical resection.

**Table 1:** Clinical features of patients with cardiac lipoma

Case number	Sex/age (year)	Clinical presentation	Lipoma location and attachment	Shape and size (cm)	Treatment	Follow-up
1	M/51	Exertional dyspnea and chest distress for 1 year	LV, attached to the anteroseptal endomyocardium	Round, 3.8 × 2.5	Surgical resection	Well after 83 months
2	F/52	Dyspnea for 1 month and a history of giant pericardial lipoma partial resection	Intrapericardial, infiltration into the biventricular myocardium	Irregular, 9.6 × 8.9	Observation	Well after 68 months
3	F/58	Incidental finding in CT for epigastric discomfort	RV, attached to the free wall of RV	Elongated, 1.9 × 1.2	Observation	Well after 49 months
4	M/56	Incidental finding in coronary artery CT for CAD	Intrapericardial, attached to the epicardium of LV	Round, 3.0 × 2.1	Observation	Well after 42 months
5	M/82	Incidental finding in checkup for facial paralysis	LV, attached to the apical lateral endomyocardium	Round, 1.5 × 1.2	Observation	Well after 40 months
6	F/34	Incidental finding in echocardiogram for paroxysmal supraventricular tachycardia	LV, attached to the apical inferior endomyocardium; RV, attached to IVS	Round, 1.5 × 1.1(LV); Round, 1.1 × 0.8 (RV)	Observation	Well after 40 months
7	M/45	Incidental finding in CT for chest trauma	LV, attached to the inferior mid-apical endomyocardium	Round, 2.0 × 1.5	Observation	Well after 35 months
8	M/64	Incidental finding in preoperative examination for urinary calculi	RA, attached to the roof of RA	Round, 2.4 × 2.1	Observation	Well after 17 months
9	M/65	Incidental finding in chest CT for lung cancer	Within mid-IVS	Nodular, 1.8 × 0.7	Observation	Well after 15 months
10	M/18	Incidental finding in health checkup	LV, attached to the anterolateral papillary muscle	Elongated, 1.6 × 0.4	Observation	Well after 6 months
11	M/49	Incidental finding in a preoperative examination for biliary stones	LV, attached to the inferior endomyocardium of LV apex	Heart-shaped, 2.5 × 2.1	Surgical resection	Well after 5 months
12	F/52	Incidental finding in health checkup	Intrapericardial	Irregular, 10.8 × 7.9	Surgical resection	Well after 3 months

*F* Female, *IVS* interventricular septum, *LV* left ventricle, *LVOT* left ventricular outflow, *M* male, *RA* right atrium, *RV* right ventricle

Most patients had only one solitary cardiac lipoma except for patient 6 in which two lipomas were found separately in bilateral ventricles. Cardiac lipomas in most (10, 83.3%) patients were found incidentally during health checkup or examination for other purposes. One patient was referred to echocardiography for exertional dyspnea and chest distress, which was found to be a result of obstruction of the left ventricular outflow by a cardiac mass. Of note, another patient with a history of cardiac lipoma resection and having dyspnea for 1 month showed a pericardial mass in echocardiography.

**Fig. 1:**
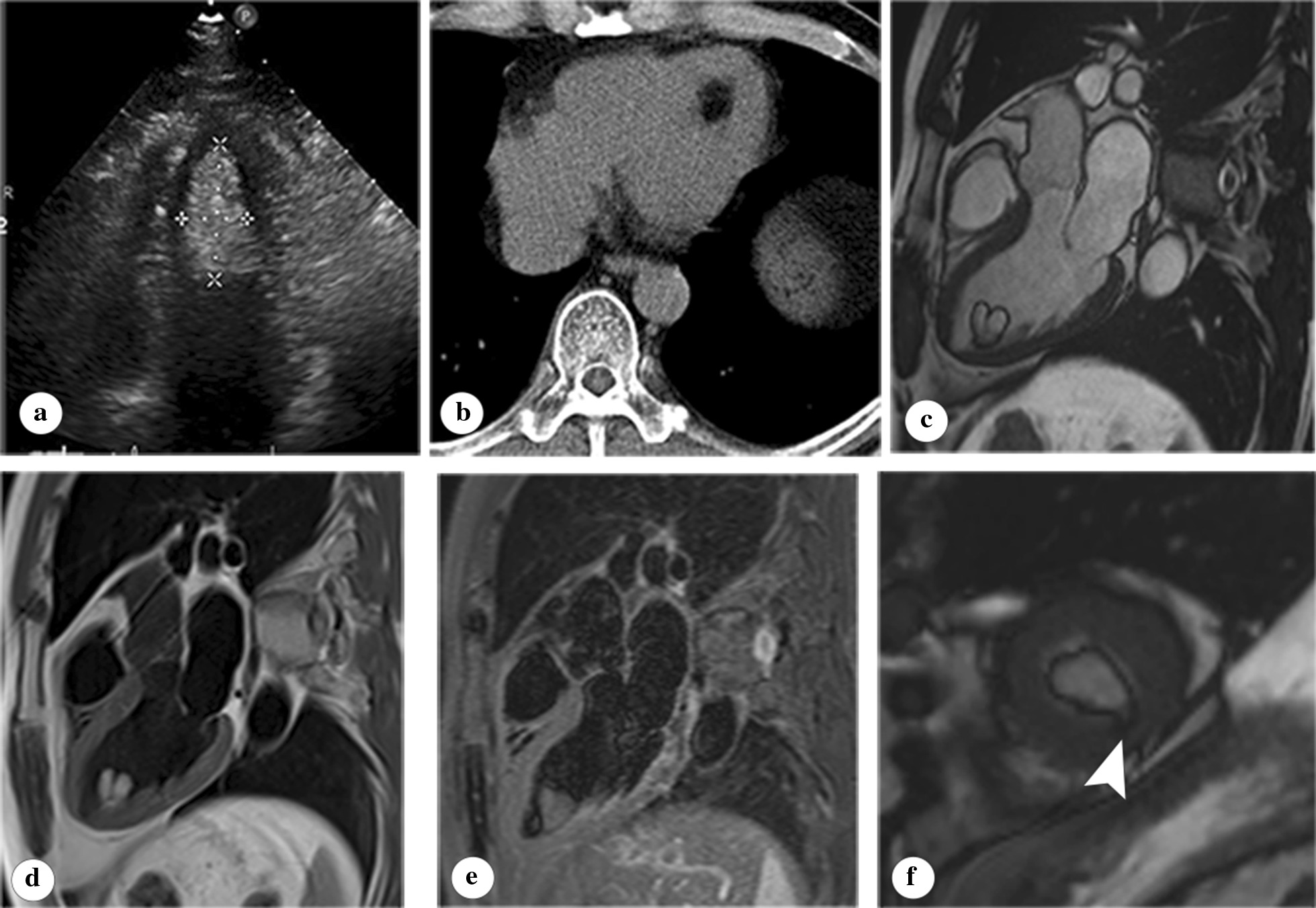
Images of a ventricular lipoma in an asymptomatic male patient (part of pictures have been previously published in the review by our griup [13]). The ventricular lipoma presented as a well-defined homogenous hyperechoic mass within left ventricle (**a**). The lipomas showed homogenous low density (-100 HU) on CT (**b**). On cardiac MRI, the lovely heart-shaped lipoma showed same signal intensity with subcutaneous fat in all sequences including cine sequence (**c**), T1 weighted image (**d**), T2 weight fat suppression image (**e**). Particularly, the narrow pedicle of the lipoma was depicted in short axis image attached to the endomyocardium (**f**, arrowhead)

The location of included cardiac lipomas showed a wide distribution across the heart. Nine of 13 (69.2%) lipomas were located within the chambers of the heart, 3 (23.1%) within the pericardium, and 1 (7.7%) within the interventricular septal myocardium (Table 1). Among masses in cardiac cavities, 6 (46.2%) were in the left ventricle, 2 (23.1%) in the right ventricle, and 1 (7.7%) in the right atrium. Particularly, one lipoma was found to originate from the anterolateral papillary muscle of the left ventricle. All lipomas within cardiac chambers were sessile except for a narrow pedicle attachment of lipoma within the left ventricle in patient 11 (Fig. 1). Lipomas within the pericardium presented as a mass with a relatively small size or an irregular extension with a larger size. Of the included cases, lipomas within the cardiac chamber had a much smaller size compared with those within the pericardium; the size of the former ranged from 1 to 4 cm, whereas the latter’s size even exceeded 10 cm.

Noninvasive cardiac imaging methods were important in the detection and diagnosis of cardiac lipomas (Table 2). Echocardiography was performed as the first-line screening method in most patients, and nine lipomas were detected with echocardiography. Most lipomas showed a well-defined border and homogenous echo intensity, except for heterogeneous echo shown in the giant recurrent pericardial mass of patient 2. The nature of cardiac masses could not be determined in all cases.

**Table 2:** Imaging findings of included cardiac masses

Case number	Echocardiographic finding	CT finding	MRI findings		
			T1W/T2W/Fat sat	T1/T2 value (ms)	Post-Gd
1	Hyperechogenic LV mass	–	Hyper/hyper/hypo	–	–
2	Heterogeneous pericardial mass	–	Hyper/hyper/hypo	–	–
3	Unremarkable	–	Hyper/hyper/hypo	–	No enhancement
4	Unremarkable	Homogenous hypodense mass (–87 HU)	Hyper/hyper/hypo	215/64	
5	Hyperechogenic LV mass	–	Hyper/hyper/hypo	220/62	-
6	Undetermined LV mass	–	Hyper/hyper/hypo	245/71	No enhancement
7	Hyperechogenic LV mass	Homogenous hypodense mass (–45 HU)	Hyper/hyper/hypo	244/61	No enhancement
8	Hypoechogenic RA mass,	Homogenous hypodense mass (–95HU)	Hyper/hyper/hypo	255/65	No enhancement
9	Unremarkable	Homogenous hypodense mass (–90 HU)	Hyper/hyper/hypo	–	–
10	Hyperechogenic LV mass	–	Hyper/hyper/hypo	–	No enhancement
11	Hyperechogenic LV mass	homogenous hypodense mass(-100HU),	Hyper/hyper/hypo	–	–
12	Hypoechogenic pericardial mass	Homogenous hypodense mass (–105HU)	Hyper/hyper/hypo	–	No enhancement

*HU* hounsfield unit, *hyper* hyperintense, *hypo* hypointense, *LV* left ventricle, *RA* right atrium

Chest or cardiac CT scanning performed in six patients showed homogenously low-density masses with well-defined borders. Their CT value ranged between –45 and –100 Hounsfield units (HU), which was equal to that of fat tissue. Benign lipoma was the preferred diagnosis based on the morphology and attenuation value.

**Fig. 2:**
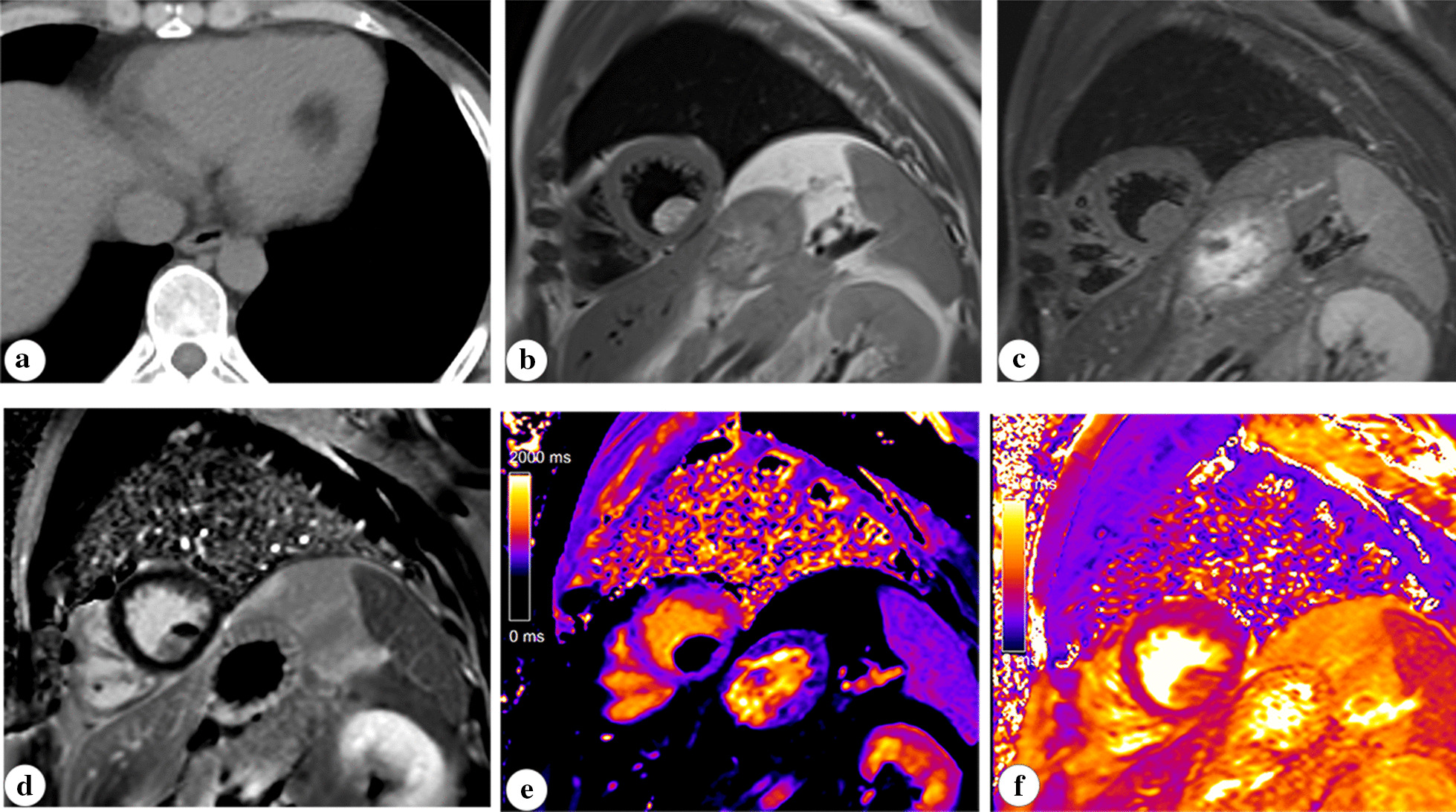
CT and cardiac MR images of a sessile ventricular lipoma in an asymptomatic male patient. The attenuation value in CT (**a**, − 45 HU) and characteristic findings in MRI (**b**, **c**, **d**) of the mass confirmed the nature of fat tissue. Besides, the ventricular mass showed same T1 (244 ms) and T2 values (61 ms) as subcutaneous fat in tissue mapping sequences (**e**, **f**)

**Fig. 3:**
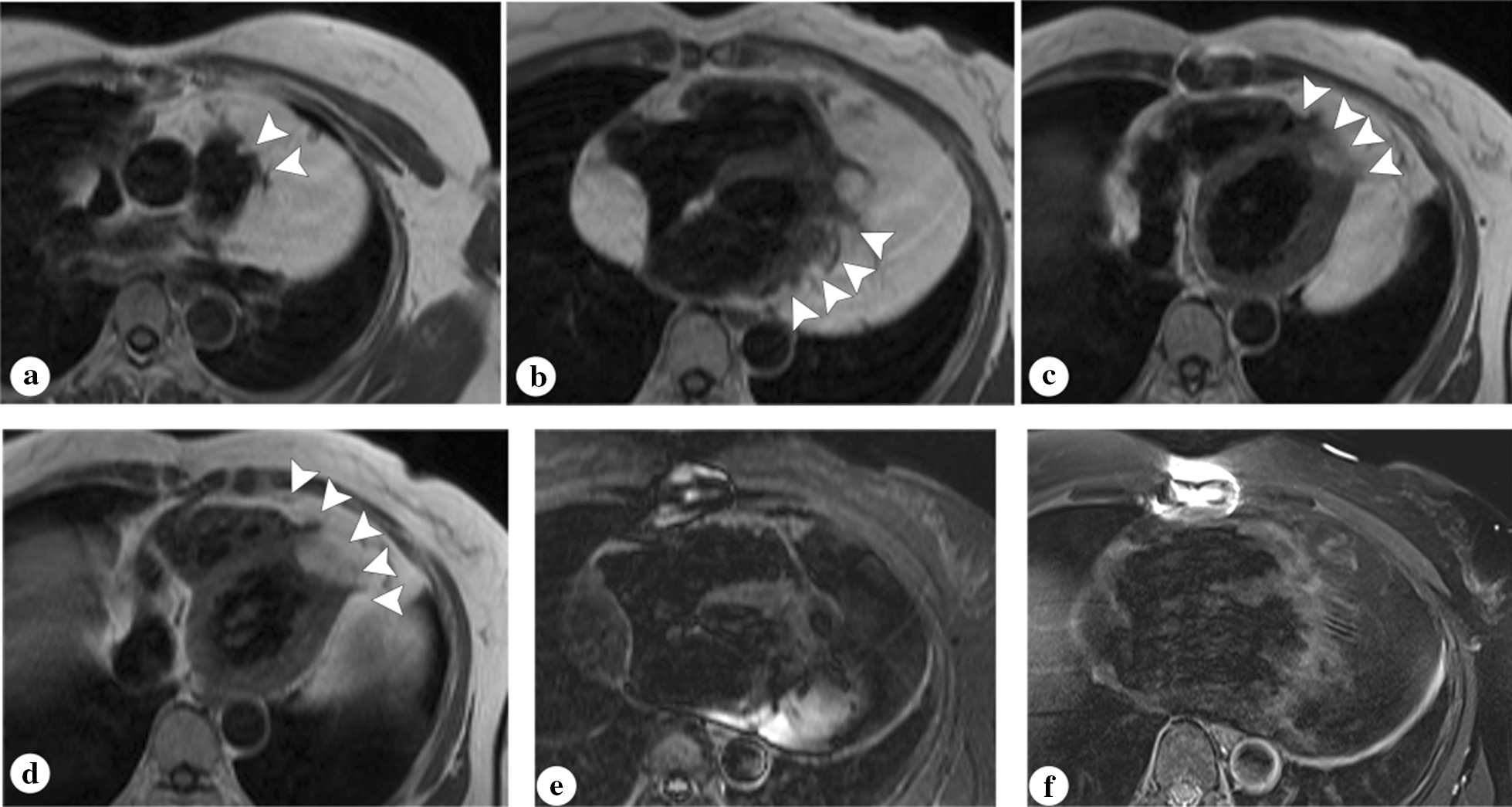
MR images of a giant pericardial lipoma of a female patient with history of pericardial lipoma resection. The T1 weighted (**a**, **b**, **c**, **d**) and T2 weighted fat suppression (**e**, **f**) images confirmed the diagnosis of lipoma. Diffuse infiltration of the lipoma to pulmonary arterial wall (**a**) and ventricular myocardium (**b**, **c**, **d**) was clearly observed (arrowhead)

**Fig. 4:**
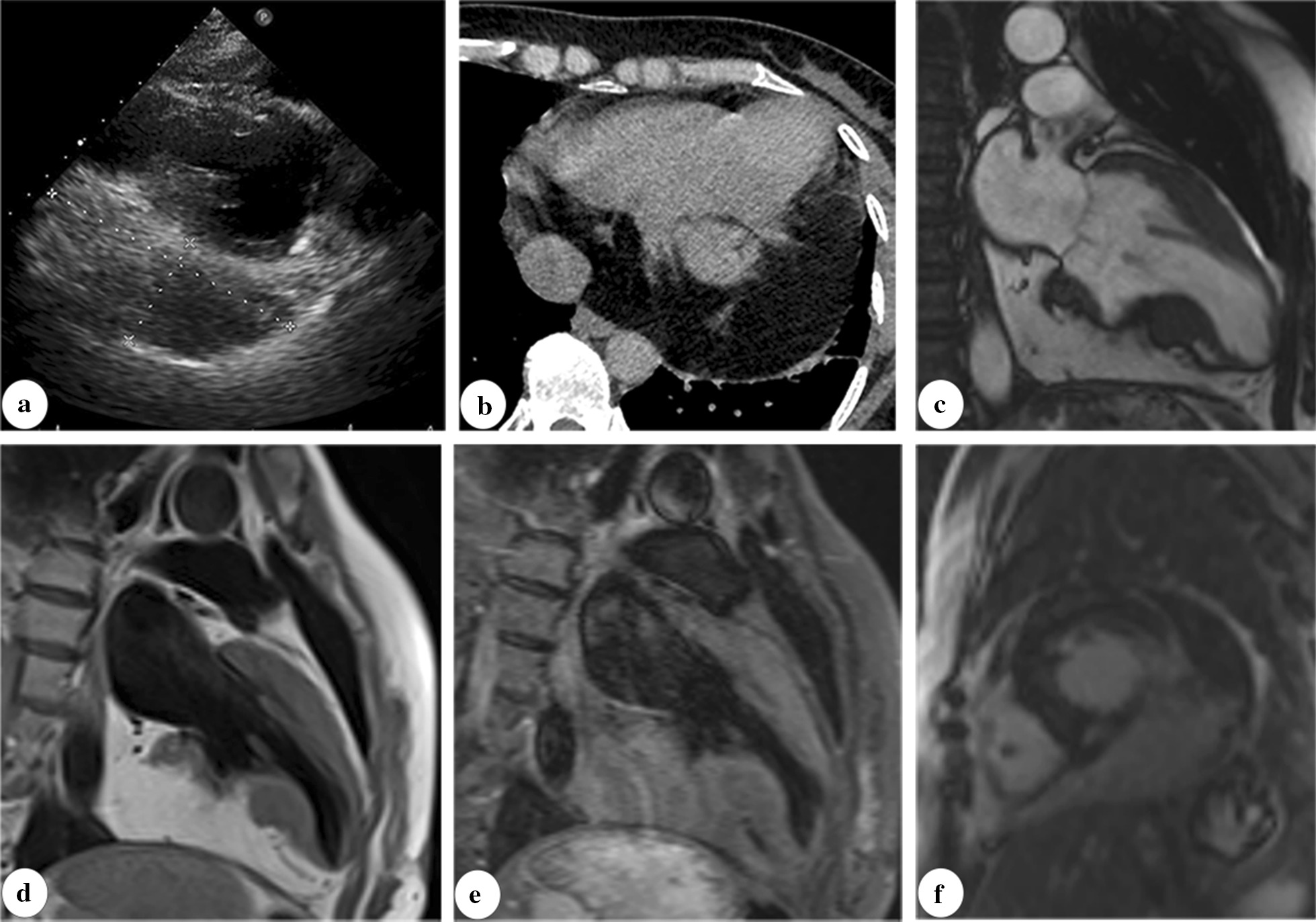
Images of a giant silent pericardial lipoma in a female patient (part of pictures have been previously published in the review by our group [13]). The giant lipoma within pericardium presented as hypoechoic mass in echocardiogram (**a**). CT image showed a homogenous low-density mass (− 105 HU) within pericardium (**b**). The MR images demonstrated a pericardial mass with same signal intensity with fat tissue (**c**, **d**, **e**, **f**). Besides, regional thinning of the inferior and apical wall of the left ventricle was observed without enhancement in the late gadolinium scanning image (**f**)

Electrocardiographically-gated cardiac MRI were available for all patients. The shape and size of all cardiac lipomas were clearly depicted in cardiac MRI. Most intracardiac lipomas had a broad base attached to the myocardium except for narrow pedicle attachment in patient 11. All lipomas showed homogenous signal features similar to that of subcutaneous fat tissue. They presented a homogenous high signal in a cine sequence with a black boundary artifact, which was more obvious for those within the cardiac chamber. A high signal was observed for all cardiac masses in T1-weighted images, and complete signal loss was seen in the fat saturation sequence. Tissue mapping images were acquired in four patients, with extremely low T1 values (200–300 ms) and high T2 values (60–80 ms) at 1.5 T, which were equal to those of the subcutaneous fat (Fig. 2). No sign of enhancement of cardiac masses was observed in patients who received contrast-enhanced scanning. Specifically, diffuse myocardial infiltration by pericardial lipoma was shown in patient 2, involving the apexes of bilateral ventricles; besides, the regional wall of the pulmonary artery root was also involved (Fig. 3). An abnormality of cardiac hemodynamics was shown in two patients with a cine sequence. Partial obstruction of the left ventricular outflow by the ventricular lipoma was clearly observed in patient 1 in the cine sequence (online video 1). The regional thinning of the inferior and apical walls of the left ventricle without late gadolinium enhancement was observed particularly in patient 12 with giant pericardial lipoma (Fig. 4). It presented as pouch protrusions in cine sequence (online video 2 and 3), showing the appearances of a pseudoaneurysm.

The surgical resection of lipoma was performed in patient 1 to relieve left ventricular outflow obstruction. Patient 2 with a giant recurrent pericardial lipoma was advised to undergo cardiac transplantation due to diffuse infiltration to the biventricular myocardium. However, the patient refused and chose to follow up periodically. Among 10 patients with silent cardiac lipoma, only 2 underwent surgical removal. Surgery was performed in patient 11 to prevent potential detachment of the lipoma because it had a narrow pedicle. In patient 12, the giant pericardial lipoma was also removed. Thinning and abnormal motion abnormality of the regional ventricular wall was thought to be the result of a gradual invasion of lipoma, and surgical resection was adopted to stop the ongoing process. The remaining eight asymptomatic patients had a regular follow-up of their cardiac lipomas. All intracardiac lipomas and the giant pericardial lipoma of operated patients were successfully resected with the assistance of extracorporeal circulation. The pathological result confirmed the preoperative diagnosis of lipoma, which was composed of mature adipose tissue.

Patients were followed up telephonically. No recurrence was reported in operated patients. The patients received conservative treatment and remained well, despite the persistence of symptoms in some of them, without the occurrence of any adverse cardiac events.

## Discussion

Lipomas are defined as mesenchymal tumors seen at a site where adipose tissue is normally present. True lipomas with encapsulation located in the heart and pericardium are very rare. The incidence of primary heart tumors reported in the autopsy series is between 0.2% and 0.4% [8, 9], of which cardiac lipomas account for 8.4% [2]. Lipomas may occur anywhere in the heart and pericardium, including cardiac valvular leaflets with the predilection of subendocardial origin [10–12]. The location of cardiac lipomas in the present study also showed predilection within cardiac chambers [13].

Despite being benign, cardiac lipomas may have a clinical presentation from mild discomfort to even syncope depending on their location and size [14]. The lipoma within the left ventricle in patient 1 in the present study caused obstruction to the outflow tract and resulted in clinical symptoms similar to those of left ventricular failure. Besides, the presence of infiltration to the myocardium by lipoma may result in severe presentation [15]. The pericardial lipoma in patient 12 remained silent despite having a giant size. However, the evident chest distress in patient 2 with pericardial lipoma of similar size might be related to diffuse infiltration to the biventricular myocardium.

Most of symptomatic cardiac lipomas can be cured by radical resection. The early detection and accurate diagnosis are of critical significance. Since the clinical application of x-ray imaging tools, the noninvasive detection of cardiac masses, including lipomas, came into realization, which was further facilitated by echocardiography. The advantages of easy availability and convenient operation make echocardiography the preferred screening tool for cardiac masses. Lipomas usually present as homogenous echogenic masses within cardiac chambers or pericardium [3, 16]. The acoustic characteristics of lipomas can help exclude cardiac malignancies [17, 18], but it is difficult to distinguish them from other benign lesions, such as myxoma, by echocardiography [19]. Fortunately, this has become possible with the application of CT and MRI [20, 21].

Lipomas have the same composition as subcutaneous fat and consist of mature adipose tissue. They have the same imaging appearance as that of subcutaneous fat in CT and MRI [22]. As shown in the present study, most cardiac lipomas can be accurately diagnosed using CT and MRI. On CT, they present as homogenous encapsulated masses with low attenuation (Hounsfield measurement < –50) [15, 23]. The signal intensity of cardiac lipomas was consistent with that of subcutaneous fat in all MRI sequences, especially the characteristic complete signal loss of the mass in fat suppression sequence [24, 25]. The application of tissue mapping in cardiac MRI made the diagnosis of lipoma more definitive [26, 27]. True cardiac lipomas are highly specific in CT and MRI; the main difference is liposarcoma. The size of the lesion and the lipomatous content are reliable discriminators between extracardiac lipomas and liposarcomas [28]. Thickened or nodular septa, associated nonadipose lesions, prominent foci of high T2 signal, and prominent areas of enhancement are important findings suspicious for liposarcoma [29]. The main differential aspects between lipoma and liposarcoma are listed in Table 3. However, the differentiation between mature cardiac lipomas and well-differentiated liposarcomas may be difficult by imaging [30]. The information extracted from radiomics may be of value in differentiating between two closely related entities [31].

**Table 3:** Main differential aspects between lipoma and liposarcoma

	Lipoma	Liposarcoma
Size	Usually small (< 10 cm)	Usually large (> 10 cm)
Margin	Well-defined and smooth	Nodular
Percentage of fat	High (> 75%)	Low (< 75%)
Septa	No or few thin	Thick or nodular
High T2 signal foci	No or minimal	Prominent
Septal enhancement	No or minimal	Prominent

Symptomatic cardiac lipomas were more likely to be resected, while silent lipomas were conservatively treated more often. However, the treatment is highly individualized. Noninvasive cardiac imaging tools including CT and MRI, especially the latter, may be important in clinical decision making regarding cardiac lipomas. Most cardiac lipomas had a broad base attached to the myocardium, while the narrow pedicle attachment, which is best observed with MRI, may indicate radical resection even in asymptomatic patients to prevent potential detachment. On the contrary, cardiac lipomas have no fixed growth pattern. Although most of them show an indolent nature [32], aggressive growth, including infiltration into the myocardium, has been reported. Cardiac lipomas may grow to a very large size and infiltrate the myocardium deeply [33, 34]. In some cases, they are even capable of melting the myocardium and developing cavities in the lipomas that communicate with cardiac chambers, thus having a pseudoaneurysmal appearance [35, 36]. With cardiac MRI, the infiltrative growth of cardiac lipomas can be clearly depicted for better clinical management [37]. As shown in patient 2 in the present study, the diffuse myocardial infiltration made the radical removal of the giant pericardial lipoma impossible. Cardiac transplantation was advised for the best solution, though refused by the patient. The imaging findings from MRI in patient 12 also significantly affected clinical decision making. Although the giant pericardial lipoma remained silent, the regional thinning of the ventricular wall, similar to the pseudoaneurysmal appearance, raised the suspicion that the lipoma gradually melted the myocardium [38, 39]. Operative resection was adopted to prevent further growth and potential melting of the ventricular wall.

In addition, cardiac imaging tools are indispensable in the follow-up of cardiac lipomas [40]. Very rarely, the recurrence of cardiac lipomas has been reported in few cases [15, 34]. However, no consensus was formed on the follow-up of simple lipomas. A regular visit with cardiac imaging is advocated for all patients undergoing resection. A more frequent follow-up in patient 12 in the present study prompted the earlier discovery of recurrence and a chance to remove the lipoma without transplantation. Patients initially not considered for surgery should be closely followed up using imaging methods because potential infiltrative growth into the myocardium may result in failed resection in the future [33]. On the contrary, despite no evidence that cardiac lipoma might undergo malignant transformation, mature lipomas and well-differentiated liposarcomas coexist in one heart. A regular review of the tumor would be the best choice, especially using MRI.

## Conclusions

Cardiac lipoma is an uncommon primary cardiac tumor. Early detection and accurate diagnosis can be achieved with noninvasive imaging tools, especially MRI. Moreover, the findings from imaging may affect clinical management through a comprehensive evaluation of the morphology of lipoma and the involvement of adjacent structures.

## Data Availability

All data generated or analyzed during this study are included in this published article.

## Abbreviations

CT: Computed tomography
MRI: Magnetic resonance imaging
HU: Hounsfield unit
